# Systemic Digital Exploitation and Counteractive Persuasive Technologies: intermediate-level concept synthesis from an annotated portfolio

**DOI:** 10.3389/frai.2026.1952265

**Published:** 2026-10-13

**Authors:** Kirill Kronhardt, Jens Gerken

**Affiliations:** Inclusive Human-Robot Interaction, Department of Rehabilitation Sciences, TU Dortmund University, Dortmund, Germany

**Keywords:** algorithmic profiling, counteractive persuasive technology, dark patterns, deceptive patterns, information disorder, misinformation, systemic digital exploitation

## Abstract

While persuasive technologies traditionally aim to drive positive behavioral changes, modern digital ecosystems increasingly weaponize persuasion to harm users through phenomena like Deceptive Patterns, Algorithmic Profiling, and Information Disorder. This covert exploitation undermines user autonomy and the fundamental right to mental self-determination. As legal frameworks to address these threats take time to implement, we approach the problem through a proactive technological lens. In this hypothesis and theory paper, we present an annotated portfolio comprising mitigation tools (*n* = 3) from our prior applied research, sharing a common design lineage (a deceptive pattern resistance game, a privacy transparency tool, and a proactive Socratic nudging tool). Based on the structural similarities we identified across these tools and the phenomena they counter, we propose two concepts to help guide future design and research: Systemic Digital Exploitation (SDE) and Counteractive Persuasive Technologies (CPT). SDE hypothesizes that these (and similar) disparate digital harms share the structural similarity of exploiting psychological vulnerabilities to create a Knowledge-Behavior Gap, bypassing rational decision-making. In response, CPTs actively deploy persuasive strategies to neutralize SDE, bridging the Knowledge-Behavior Gap to restore user autonomy and scaffold mental self-determination. We arrive at these frameworks through a retrospective analysis of our prior work, presenting no new empirical data.

## 1 Introduction

A growing body of Human-Computer Interaction (HCI) research addresses digital phenomena that actively and negatively impact users (Gray et al., 2024a; Lutz et al., 2025; Arias-Cabarcos et al., 2023). Modern digital environments have increasingly evolved from neutral tool sets into highly optimized, adversarial ecosystems. Phenomena such as *Deceptive Patterns* in interface design, *Algorithmic Profiling* in the privacy domain, and *Information Disorder* in news and social media weaponize behavioral psychology to extract consent, information, or approval from users where they might otherwise not have acted accordingly. Each of these harms has matured into a distinct sub-discipline, developing its own specialized taxonomies (Gray et al., 2024b; Norberg et al., 2007; Wardle and Derakhshan, 2017) and interactive countermeasures (Wallerius et al., 2025; Schufrin et al., 2020; Hasimi and Poniszewska-Marńda, 2023). However, approaching these and other digital harms through isolated, domain-specific lenses may obscure overarching structural mechanisms, limiting our theoretical understanding of how user autonomy is fundamentally undermined across the digital landscape.

Simultaneously, the ways in which technology can be designed to shape human behavior have been extensively formalized within the domain of Persuasive Technologies (PT). Utilizing established frameworks such as Fogg's Behavior Model (FBM) (Fogg, 2009) and the Persuasive Systems Design (PSD) model (Oinas-Kukkonen and Harjumaa, 2009), interactive systems deliberately employ strategies like nudging, gamification, and social proof to alter user behavior. Historically, PTs have been deployed to drive positive, system-defined behavior changes in domains such as health (Halko and Kientz, 2010; Fritz et al., 2014), clinical adherence (Kelders et al., 2012), and sustainability (Adaji and Adisa, 2022). To achieve behavioral change, PTs may rely on bypassing reflective decision-making, leveraging automatic System 1 processing and routine reward-seeking (Smids, 2012; Fritz et al., 2014) to nudge users toward the system architect's predetermined objective (Purpura et al., 2011; Wenker, 2022).

As digital environments grow increasingly hostile, this standard, paternalistic approach to persuasive technology reveals ethical and functional limitations. Recent systematic literature reviews explicitly critique systems that enforce unilaterally defined targets, warning that such approaches risk overriding user agency and devolving into soft paternalism or psychological manipulation (Rahman and Adaji, 2024; Wenker, 2022). In the terminology of Self-Determination Theory (SDT) (Ryan and Deci, 2000), standard PTs often build on external regulation. The academic community is increasingly recognizing these risks, prompting efforts to formalize the ethical boundaries of PT and identify when persuasion crosses into manipulation. For instance, (Nyström and Stibe 2020) emphasize the urgency of classifying harmful designs, concluding that there is a critical need to continuously explore and monitor emerging forms of “dark” PT to refine our theoretical understanding.

Furthermore, in a society increasingly saturated with artificial intelligence and computational persuasion, legal and ethical scholars argue that defending against manipulation requires formally recognizing an individual's fundamental right to “mental self-determination” (Faraoni, 2023). Because adaptive algorithms can covertly bypass rational decision-making with unprecedented precision, scholars like Faraoni (2023) argue that existing rights to privacy or freedom of expression are insufficient; while traditional rights protect the *expression* of a thought, individuals must now be explicitly protected in their capacity to *create* thoughts free from algorithmic “hypernudging” (Faraoni, 2023). However, responding to such calls may require moving beyond merely identifying adversarial environments or defining legal boundaries. When users navigate ecosystems characterized by increasing hostility, the mitigative goal is not to nudge them toward a new, system-defined behavior, but rather to actively defend their baseline psychological need for autonomy. A critical theoretical gap therefore exists regarding how interactive systems can be designed not to impose a “good” behavior, but to actively counteract pre-existing environmental manipulation.

While formalizing legal rights is a vital step, regulation and its actual deployment are often notoriously slow and may present with legislative loopholes when confronting rapidly evolving technologies. This practical reality motivates a technological response through an HCI lens: rather than waiting solely for top-down legal enforcement, we can proactively design supportive systems that defend users against being “hypernudged out of their mental self-determination” (Faraoni, 2023). To address the theoretical gap of how interactive systems can practically scaffold this autonomy, this hypothesis and theory paper presents a retrospective analysis of our recent applied research in PTs.

Initially, our mitigative efforts did not stem from a unified theory, but rather from the independent design, development and evaluation of three distinct interactive tools targeting isolated digital harms: a serious game (*Trickery*) addressing the interface-level manipulations of Deceptive Patterns (Kronhardt et al., 2024b); a visualization tool (*PERSONÆR*) addressing the infrastructural opacity of Algorithmic Profiling (Kronhardt et al., 2024a); and a proactive Socratic nudging tool (*FallacyCheck*) addressing the cognitive challenges of Information Disorder (Kronhardt et al., 2025b).

Following their individual evaluations, we sought to contextualize our practical design outcomes within the broader discourse on ethical PT and user agency. Methodologically drawing upon Research through Design (RtD) (Zimmerman et al., 2007), we first compile these tools into an annotated portfolio (Bowers, 2012) to facilitate a *post-hoc* reflexive analysis. Through this retrospective lens, we observe structural similarities both in the way PTs themselves may counteract digital harms, and in the underlying structures that enable those harms in the first place. We hypothesize that these (and other) digital harms share a structural similarity: the active exploitation of a *Knowledge-Behavior Gap*. By that, we refer to a strategically induced rift between a user's reflective knowledge of a risk and their actual in-the-moment behavior, designed to bypass reflective decision-making. We tentatively formalize this structural similarity as *Systemic Digital Exploitation (SDE)*. To directly counteract SDE and address the need for non-paternalistic defense mechanisms, we propose *Counteractive Persuasive Technologies (CPT)*. We posit that effective interventions in hostile environments may need to evolve from passive transparency toward active scaffolding and capacity-building. By deploying persuasive strategies to disrupt automaticity and buy users cognitive bandwidth, instead of paternalistically defining a “good” behavioral outcome, CPTs aim to bridge the Knowledge-Behavior Gap and facilitate integrated regulation (Ryan and Deci, 2000). We thus offer a foundation for future empirical work on restoring digital autonomy and a provisional unifying lens to view these disparate phenomena of digital harm across their individual research streams.

In summary, we make the following theoretical and methodological contributions:

- A retrospective, cross-domain *annotated portfolio* of three mitigation tools, tracing structural similarities across interventions in distinct research silos (Deceptive Patterns, Algorithmic Profiling, Information Disorder).
- The initial formalization of *Systemic Digital Exploitation (SDE)*, which defines the strategic design of digital environments to actively exploit human psychological vulnerabilities, bypassing rational decision-making to induce a Knowledge-Behavior Gap and thwart user autonomy.
- The initial formalization of *Counteractive Persuasive Technology (CPT)*, which outlines how digital interventions can actively deploy persuasive strategies to counteract SDE, bridging the Knowledge-Behavior Gap to restore user autonomy and support mental self-determination.

The remainder of this paper is structured as follows. Section 2 establishes the theoretical background, detailing PTs, the associated ethical gap in adversarial environments, and the methodological background of RtD (Zimmerman et al., 2007). Section 3 introduces the annotated portfolio of three interactive mitigation tools and corresponding digital harms from our prior applied research. Section 4 synthesizes these cross-domain observations to propose the concepts of SDE and CPT. Section 5 discusses the boundary conditions of SDE and presents the CPT design process, showing how its adaptation of established persuasive design frameworks addresses the ethical tension of soft paternalism. Finally, Section 6 discusses limitations and Section 7 concludes with pathways for future empirical validation.

## 2 Related work

### 2.1 Persuasive Technologies

PTs are defined as interactive computing systems purposefully designed to alter users' attitudes or behaviors (Fogg, 2009). Their primary utility lies in the technological “automation” of behavior change, effectively bridging the gap between passive tool usage and active behavioral adaptation. To formalize how technology can reliably drive this behavior change, Fogg (2009) introduced FBM. FBM postulates that a target behavior only occurs when three specific elements converge simultaneously: Motivation, Ability, and a Prompt (*B* = *MAP*). Motivation is categorized into three core drives: pleasure/pain, hope/fear, and social acceptance/rejection. Ability, conversely, is framed not as teaching users new skills, but as maximizing “simplicity.” Simplicity is determined by the user's scarcest resource at the moment of action, constrained by factors such as time, money, physical effort, cognitive load (brain cycles), social deviance, and routine. Finally, prompts initiate the behavior and are deployed based on the user's specific cognitive state: “Sparks” for low motivation, “Facilitators” for low ability, and “Signals” when both motivation and ability are sufficiently high.

While models like the FBM explain the psychological prerequisites for behavior change, they lack systematic analysis and design methodologies required for actual software engineering (Oinas-Kukkonen and Harjumaa, 2009). To bridge this gap, Oinas-Kukkonen and Harjumaa (2009) developed the PSD framework, detailing how to conceptualize, develop, and analyze persuasive software. The PSD framework is built upon seven underlying postulates. These assert that information technology is never truly neutral, that people want an organized and consistent worldview, and that both direct and indirect routes are key persuasion strategies. Moreover, persuasion is often incremental, and must be transparent, unobtrusive, and useful.

The PSD framework operationalizes persuasive design through two main phases. First, designers must rigorously analyze the persuasion context, which encompasses:

- **The Intent:** Identifying whether the persuader is endogenous (system creators), exogenous (distributors), or autogenous (the users themselves), and determining if the target is an attitude or behavior change.
- **The Event:** Analyzing the specific use case, user characteristics (goals, motivations), and technological constraints.
- **The Strategy:** Defining the message (conviction via logic vs. persuasion via emotion) and the route (direct cognitive evaluation versus indirect heuristic cues).

Second, designers map system qualities across four functional categories to execute the persuasion strategy (Oinas-Kukkonen and Harjumaa, 2009):

- **Primary Task Support:** Features that reduce complexity and guide users, such as tunneling, personalization, reduction, and simulation.
- **Dialogue Support:** Mechanisms that facilitate computer-human interaction, including praise, reminders, rewards, and visual attractiveness (liking).
- **System Credibility Support:** Elements that foster trust, such as demonstrable expertise, verifiability, authority, and a “real-world feel.”
- **Social Support:** Features leveraging interpersonal dynamics, such as normative influence, social learning, competition, and recognition.

In practice, the operationalization of these PSD principles has historically relied on deploying PTs to drive positive, system-defined behavior changes. Consequently, PSD has seen widespread application in domains such as health promotion. In this field, applications tailor persuasive strategies to users' Big Five personality traits (Halko and Kientz, 2010) or rely on wearable sensors and continuous self-monitoring to motivate physical activity (Fritz et al., 2014). Similarly, PSD elements like dialogue support (e.g., automated reminders) are heavily utilized to increase patient adherence to clinical web-based interventions (Kelders et al., 2012). Furthermore, sustainability interventions deploy rewards and suggestions through mobile apps and IoT devices to drive eco-friendly habits like energy conservation (Adaji and Adisa, 2022).

However, as these systems mature, they are increasingly utilized to optimize compliance to external targets rather than foster internal volition. For example, recent reviews highlight the pervasive use of PTs and gamification in the digital workplace to align employee behavior directly with an employer's organizational agenda. This alignment is often achieved by framing productivity metrics and workflow constraints as game-like achievements (Wenker, 2022). To achieve these system-architected objectives, modern PTs frequently leverage primary task and dialogue support (e.g., tunneling, gamification, and continuous feedback) to bypass reflective decision-making, relying instead on automatic System 1 processing and routine reward-seeking (Smids, 2012; Fritz et al., 2014). This dynamic is vividly illustrated by the phenomenon of “number fishing” in fitness trackers, where users perform meaningless tasks (like walking in circles inside their house) solely to activate system rewards. This practice effectively supplants their underlying health motivations with the system's artificial metrics (Fritz et al., 2014). Furthermore, persuasive systems can sometimes deplete a user's self-regulatory capacity (e.g., via persistent, un-mutable warnings) or operate entirely outside their conscious awareness (such as through subliminal feedback). When this occurs, they cross the ethical boundary from voluntary persuasion into manipulation or coercion (Smids, 2012). This prevailing trend establishes a paradigm of soft paternalism, where systems enforce a designer's predetermined, rationalized objective—dictating the “one right way” to behave—rather than scaffolding the user's autonomous choices (Purpura et al., 2011).

### 2.2 Ethical concerns and moving toward the right to mental self-determination

This escalating tension between designer-enforced objectives and user autonomy underscores why the ethical implications of PTs remain a critical point of contention (Rahman and Adaji, 2024). Early critiques of PTs warned against this exact designer-centric focus that ignores unintended behavioral consequences, a phenomenon termed “compusuasion” (Atkinson, 2006). Atkinson (2006) argued that for persuasion to remain ethical and distinct from propaganda or manipulation, the designer's intent must be transparently exposed at the commencement of the user's engagement. This transparency ensures the user retains the agency to accept or reject the intervention.

However, as PTs proliferate beyond individual self-help applications into collective and ambient environments, maintaining this transparent, voluntary consent becomes increasingly complex (Timmer et al., 2015). For example, when PTs are deployed in the workplace (e.g., wellness trackers or fleet monitoring), the goals of the system provider (the employer) and the user (the employee) may diverge (Timmer et al., 2015). In such contexts, inherent power asymmetries compromise the voluntariness of the system, risking a shift from persuasion to coercion (Timmer et al., 2015). Furthermore, the integration of PTs into “smart” ambient environments enables interventions that operate below the threshold of conscious awareness. This fundamentally limits the user's capacity for critical reflection and deliberate consent (Timmer et al., 2015). Recent systematic reviews confirm that factors such as autonomy, consent, data privacy, and the use of addictive or deceptive design strategies are paramount in determining the ethical perception of these systems (Rahman and Adaji, 2024).

These evolving technological capabilities underscore the urgency of rethinking digital rights. In a comprehensive theoretical and legal review, Faraoni (2023) analyzes how AI-driven PTs escalate traditional nudging into “computational manipulation.” Faraoni (2023) notes that these systems leverage Big Data to dynamically map and exploit an individual's specific cognitive biases (System 1 processing). Consequently, they continuously reconfigure digital choice architectures in real-time—a practice termed “hypernudging” (Faraoni, 2023). Reviewing current international and European legal frameworks (e.g., the Digital Services Act and the Artificial Intelligence Act), Faraoni (2023) argues that while existing laws address data protection and informational self-determination, they fall short of addressing the core psychological threat. Traditional rights safeguard the privacy of a physical/digital space and the freedom to *express* an existing thought. In contrast, hypernudging covertly interferes with the very *creation* and formation of that thought by bypassing rational decision-making. Tracing the concept back to Kantian autonomy, Faraoni (2023) concludes that society must formally recognize a new, distinct “right to mental self-determination.” This right guarantees cognitive sovereignty, explicitly protecting the human mind from unperceived algorithmic interference. Establishing this fundamental right provides the ethical and legal mandate to shift persuasive design away from subliminal coercion and toward the active, transparent scaffolding of user autonomy.

### 2.3 Self-Determination Theory

Operationalizing this ethical mandate to protect mental self-determination and actively scaffold user autonomy requires an actionable, robust psychological foundation. SDT provides exactly this, serving as a macro-psychological framework of human motivation and personality development (Ryan and Deci, 2000). Traditional PTs often rely on behaviorist models, which view individuals as passively reacting to external stimuli or merely seeking to minimize cognitive load. In contrast, SDT posits that humans are inherently curious, vital, and proactive (Ryan and Deci, 2000). According to SDT, individuals naturally strive to master new skills and integrate experiences, provided their social and environmental conditions support this growth.

At the core of this framework is Basic Psychological Needs Theory (BPNT), a mini-theory which asserts that human flourishing depends on the fulfillment of three innate psychological needs (Ryan and Deci, 2000):

- **Autonomy:** The need to self-initiate and self-regulate one's actions, experiencing a sense of volition and inner endorsement. Crucially, autonomy does not equate to total independence or detachment from others, but rather the feeling of psychological freedom to act in accordance with one's integrated values.
- **Competence:** The need to experience efficacy and mastery in interacting with the environment. This is supported by presenting “optimal challenges”—tasks perfectly calibrated to avoid both under- and over-stimulation—and providing effectance-promoting, constructive feedback.
- **Relatedness:** The need to feel securely connected to, and valued by, others within a community. A safe and empathetic relationship often serves as the necessary foundation for users to adopt initially external behaviors.

While BPNT defines the psychological nutrients necessary for motivation, Organismic Integration Theory (OIT)—another mini-theory within SDT—provides a nuanced continuum of how behavior is regulated (Ryan and Deci, 2000). OIT challenges the binary distinction between intrinsic motivation (doing an activity purely for inherent joy) and extrinsic motivation (acting for external rewards or to avoid punishment). Instead, OIT illustrates how external regulations can be progressively internalized. This progression moves from *external regulation* (compliance), to *introjected regulation* (acting out of guilt or ego), to *identified regulation* (consciously valuing the action), and finally to *integrated regulation* (fully assimilating the behavior into one's self-concept).

Within the HCI and PT communities, SDT has increasingly been adopted as a theoretical lens to design for sustained engagement (Alberts et al., 2024; Gerstenberg et al., 2025). However, recent reviews reveal a critical translational challenge: HCI research frequently applies SDT superficially, focusing almost exclusively on fostering intrinsic motivation through gamification (Alberts et al., 2024). This approach attempts to make digital interactions inherently “fun” or enjoyable to optimize engagement with the technology itself (Alberts et al., 2024).

As Gerstenberg et al. (2025) and Alberts et al. (2024) argue, this engagement-focused approach is fundamentally limited, particularly in health and behavior change contexts where target behaviors are rarely intrinsically enjoyable. Relying purely on the enjoyment of the technology risks creating a fragile dependency; once the novelty wanes or the tool is removed, the motivation for the underlying behavior collapses (Alberts et al., 2024). Instead, researchers advocate for an *internalization-focused* approach grounded in OIT (Gerstenberg et al., 2025; Alberts et al., 2024). PTs can utilize technology as a transitional scaffold rather than an engagement trap. By doing so, they support the fulfillment of basic psychological needs to help users internalize the ultimate value of a behavior. This approach moves users along the continuum from controlled compliance toward true, integrated self-determination (Gerstenberg et al., 2025).

To ensure that persuasive features genuinely foster this internalized regulation rather than masking external compliance, SDT can also serve as a critical auditing tool (Tikka et al., 2025). To demonstrate this, Tikka et al. (2025) conducted a *post-hoc* analysis of a mobile health application designed for micro-entrepreneurs, formally cross-referencing its specific software features with the basic psychological needs those features were intended to support. By explicitly mapping PSD features (e.g., self-monitoring, reminders, praise) against operationalized SDT elements, they traced how theoretical intervention goals were executed through the user interface. Such systematic mapping helps developers dismantle the “black box” of behavioral interventions, where the actual delivery mechanisms of a theory are often obscured. Without this rigorous alignment, systems risk failing to adequately support basic psychological needs. This leaves users vulnerable to interface mechanics that inadvertently prioritize system-defined compliance over genuine autonomy.

### 2.4 Research through Design and intermediate-level knowledge

To bridge the gap between abstract psychological theory and concrete interface design, this paper aligns with the tradition of RtD (Zimmerman et al., 2007). As Zimmerman et al. (2007) outline, rather than merely producing commercial products, RtD approaches complex problems by producing design artifacts that embody and test theoretical propositions. However, a core epistemological challenge within RtD has been abstracting knowledge from specific material artifacts without forcing that knowledge into rigid, generalized scientific theories (Gaver, 2012).

To address this, the HCI community has developed the concept of *intermediate-level knowledge*, a form of knowledge that resides in the theoretical space between particular design instances and universal, grand theories (Höök and Löwgren, 2012). Much of this intermediate-level knowledge is generative, capturing design insights so they can be appropriated (Höök and Löwgren, 2012). However, the space also encompasses evaluative forms of intermediate-level knowledge aimed at theoretical sense-making (Stolterman and Wiberg, 2010; Dalsgaard and Dindler, 2014). Our annotated portfolio of three specific mitigation tools for digital harms is fundamentally an exercise in generating this intermediate-level knowledge.

Over the past decade, several constructs have been developed to structure and communicate intermediate-level knowledge. Most pertinent to our retrospective methodology is the *annotated portfolio* approach (Bowers, 2012). Instead of analyzing a single design artifact in isolation, the annotated portfolio approach collects a curated set of designs and links them through textual annotations (Bowers, 2012). These annotations articulate the “family resemblances” across the artifacts, mapping shared qualities and design decisions (Bowers, 2012). This allows researchers to capture and communicate design thinking in a descriptive and inspirational manner (Bowers, 2012). Consequently, this avoids the reductionism of forcing design practice into standardized scientific metrics (Bowers, 2012).

This abstraction process frequently yields different forms of intermediate-level knowledge, such as what (Höök and Löwgren 2012) term “strong concepts”—generative design elements or principles that are abstracted from specific instances of use. Strong concepts focus on the “dynamic gestalt,” or interactive behavior, acting as reusable design heuristics (Höök and Löwgren, 2012).

However, when the primary objective shifts from generating practical design solutions to advancing HCI theory, researchers develop *conceptual constructs* (Stolterman and Wiberg, 2010). Stolterman and Wiberg (2010) define a conceptual construct as an intellectual or logical construction that serves as a midway point between a single concept and an all-encompassing theory. Rather than optimizing for a specific problem or user situation, conceptual constructs bring together various conceptual elements and earlier findings to make sense of a complex, evolving design space. As Dalsgaard and Dindler (2014) note, conceptual constructs are often driven by a top-down intent to enrich the theoretical foundations of the field. This approach organizes disparate phenomena into a cohesive understanding.

In-between strong concepts and conceptual constructs, *bridging concepts* explicitly navigate the dual space of theory and practice (Dalsgaard and Dindler, 2014). While conceptual constructs build upward toward theory, and strong concepts build downward toward practice, bridging concepts are distinguished by their ability to facilitate exchange in *both* directions. Dalsgaard and Dindler (2014) define a bridging concept as being composed of three necessary constituents: a firm theoretical grounding, a set of design articulations (parameters that designers can manipulate to change the interaction), and a range of exemplars that demonstrate the concept's scope. By operationalizing abstract academic theories into practical design heuristics, bridging concepts provide the actionable tools necessary to translate theoretical insights back into applied design (Dalsgaard and Dindler, 2014).

## 3 Annotated portfolio of mitigation tools for digital harms

Building directly upon the RtD epistemology and the logic of annotated portfolios established in Section 2, we present a retrospective reflexive analysis of our team's applied research. While HCI is concerned with numerous digital phenomena, we focus our reflection on three specific digital harms we explored in prior applied work: Deceptive Patterns, Algorithmic Profiling, and Information Disorder. Our goal is not to suggest these are the only phenomena of concern, nor that they form a complete taxonomy. Rather, these phenomena serve as the context for three distinct interactive tools—Trickery (Kronhardt et al., 2024b), PERSONÆR (Kronhardt et al., 2024a), and FallacyCheck (Kronhardt et al., 2025b)—that we independently developed to target these isolated harms. By structuring these practical design outcomes as an annotated portfolio, we aim to deeply trace their structural overlaps.

### 3.1 Digital harm and artifact descriptions

To ground our annotated portfolio, we present the artifacts case by case. For each case, we first establish the theoretical and operational definitions of the harmful digital phenomenon. Following this, we detail the specific interactive mitigation tool we designed to counteract it. This structured reflection serves as the foundation from which we subsequently extract our intermediate-level knowledge.

#### 3.1.1 Deceptive Patterns and Trickery

Deceptive Patterns (also known as Dark Patterns) are malicious interface design strategies used to trick users into performing unintended and unwanted actions (Brignull et al., 2015; Conti and Sobiesk, 2010). These actions can lead to tangible harm including privacy invasion, financial loss, and cognitive burden (Mathur et al., 2021).

The efficacy of Deceptive Patterns relies heavily on the exploitation of human psychological biases and decision-making processes. Theoretical models link these manipulations to Kahneman's dual-process theory, positing that deceptive designs systematically exploit System 1 thinking—the fast, unconscious, and automatic mode of thought—while discouraging the engagement of System 2, which is slower and more rational (Bösch et al., 2016; Kahneman, 2011).

By capitalizing on this cognitive automaticity and the human need to belong (Bösch et al., 2016), deceptive interfaces manipulate users into consenting to extensive data collection or prolonged engagement. For instance, “Interface Interference” patterns utilize visual hierarchy to make the provider's preferred option (e.g., “Accept All Cookies”) the path of least resistance (Gray et al., 2024b), exploiting the users' tendency to prioritize immediate access to content over long-term privacy risks (Luguri and Strahilevitz, 2021). Research indicates that even when users are aware of these manipulations, they often struggle to identify specific instances or resist them due to resignation or a lack of perceived control (Di Geronimo et al., 2020; Bongard-Blanchy et al., 2021; Maier and Harr, 2020).

To address the cognitive automaticity exploited by Deceptive Patterns, we developed *Trickery*. *Trickery* is a first-person serious game that casts the player as a newly hired employee within a laboratory setting (Kronhardt et al., 2024b). Guided by an unreliable narrator—the “lead scientist” —the player navigates seven distinct rooms, each operationalizing a specific “gamified deceptive pattern” through concrete gameplay mechanics. Gamified deceptive patterns differ from standard, benign gamification. While standard gamification employs game elements (e.g., points, badges) to increase user engagement or enjoyment, a gamified deceptive pattern is the deliberate adaptation of a real-world manipulative concept into a game mechanic explicitly for educational or persuasive purposes (Kronhardt et al., 2024b). Rather than making the game more “fun”—a role reserved for separate “enriching game mechanics”—these patterns intentionally frustrate or deceive the player, having them experience the negative consequences of manipulation within a safe environment (Kronhardt et al., 2024b). For example, the “Nagging” pattern (Gray et al., 2024b) is operationalized as “Insistent Questioning,” where the narrator repeatedly pressures the player to surrender essential inventory items needed to complete the game. The “Aesthetic Manipulation” pattern (Gray et al., 2024b) is represented more abstractly, through a room with a lit pathway inviting the player to follow it, and unlit areas which the user could explore instead to find a shortcut (see Figure 1). Similarly, the “Sneaking” pattern (Gray et al., 2024b) is simulated by a vending machine that attempts to slip unwanted items into the player's cart.

**Figure 1:**
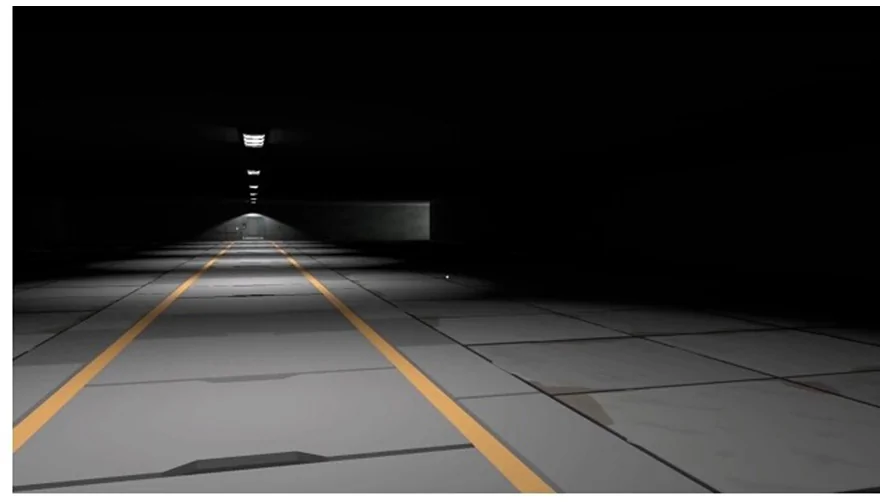
A gameplay scene from the serious game Trickery (Kronhardt et al., 2024b), operationalizing the “Aesthetic Manipulation” deceptive pattern (Gray et al., 2024b). Similar to how visually prominent buttons subtly guide users to accept broad data collection online, the game translates this interface-level manipulation into a spatial mechanic. A brightly illuminated path surrounded by darkness exploits automaticity, instinctively steering the player's navigation.

The game's design is grounded in “emotional learning,” a framework suggesting that experiencing situations with immediate consequences—such as the frustration of losing progress—results in stronger educational outcomes (Schiebener and Brand, 2015). This approach targets the psychological vulnerability of System 1 thinking; by simulating manipulation, the game triggers a cognitive shift toward System 2 thinking to identify and if necessary resist the deception (Kahneman, 2011). Furthermore, *Trickery* employs “intrinsic integration,” embedding the learning content directly into the game world rather than separating it from the narrative (Habgood and Ainsworth, 2011; Naul and Liu, 2019). This method is posited to foster deeper reflection and the creation of new neural links for understanding, contrasting with direct instruction which often only triggers short-term memory (McDrury and Alterio, 2003; Liston, 1994). Moreover, the game's persuasive mechanisms can be viewed through the lens of inoculation theory (McGuire and Papageorgis, 1961; McGuire, 1961), which posits that pre-exposure to a weakened form of an argument—or in this case, a deceptive pattern—can build resistance against stronger future attacks. By confronting players with gamified deceptive patterns in a simulated environment, *Trickery* allows users to experience the mechanics of manipulation without real-world risks.

#### 3.1.2 Algorithmic Profiling and PERSONÆR

Algorithmic Profiling describes the automated processing of personal data to evaluate aspects of a user's characteristics. Through the aggregation of a user's actively or passively shared data (Dennen, 2015; Arakerimath and Gupta, 2015), disparate and seemingly innocuous data points can be combined to create highly accurate, intimate profiles that can reveal sensitive details about a user's personality, emotions, and behavior (Merener, 2012; Insel, 2017; Goda et al., 2015).

The persistence of Algorithmic Profiling is sustained by the Privacy Paradox, a phenomenon where a significant discrepancy exists between users' stated privacy concerns and their actual disclosure behaviors (Norberg et al., 2007; Carrascal et al., 2013; Taddicken, 2014). While initially framed through Privacy Calculus Theory, assuming users rationally trade privacy for benefits (Li et al., 2010; Dinev and Hart, 2006), users typically lack the data literacy to understand the volume and sensitivity of the data collected (Farke et al., 2021; Vervier et al., 2017) and struggle to evaluate long-term ramifications against immediate gratification (Acquisti et al., 2015; Kehr et al., 2015).

Furthermore, the mechanisms of profiling are obscured by transparency tools that fail to align with human cognition. For example, in the European Union (EU), General Data Protection Regulation (GDPR) mandated data exports are often too technical for interpretation and simplified visualizations (like interest segments) rarely promote real understanding (Urban et al., 2019). This lack of actionable feedback prevents users from connecting their online actions to the resulting algorithmic profile, leaving them vulnerable to manipulation and social engineering (Pistilli et al., 2014).

To counteract the opacity and abstract nature of Algorithmic Profiling, we designed *PERSONÆR*. *PERSONÆR* is a Transparency Enhancing Tool (TET) implemented as a browser extension that visualizes a user's digital footprints narratively (Kronhardt et al., 2024a, 2025a). Functioning as an ex-post tool, it runs in the background of a user's browsing session, extracting visible text from websites and identifying significant keywords. The accumulated keyword list is transmitted to a Large Language Model (LLM), which synthesizes the data into a structured 300-word user persona. This output transforms raw browsing history into specific inferred fields—including a pseudonym, gender, age range, location, and a narrative background—thereby simulating how third-party trackers might interpret the user's passive digital behavior. An example of such an output as presented in PERSONÆR is found in Figure 2.

**Figure 2:**
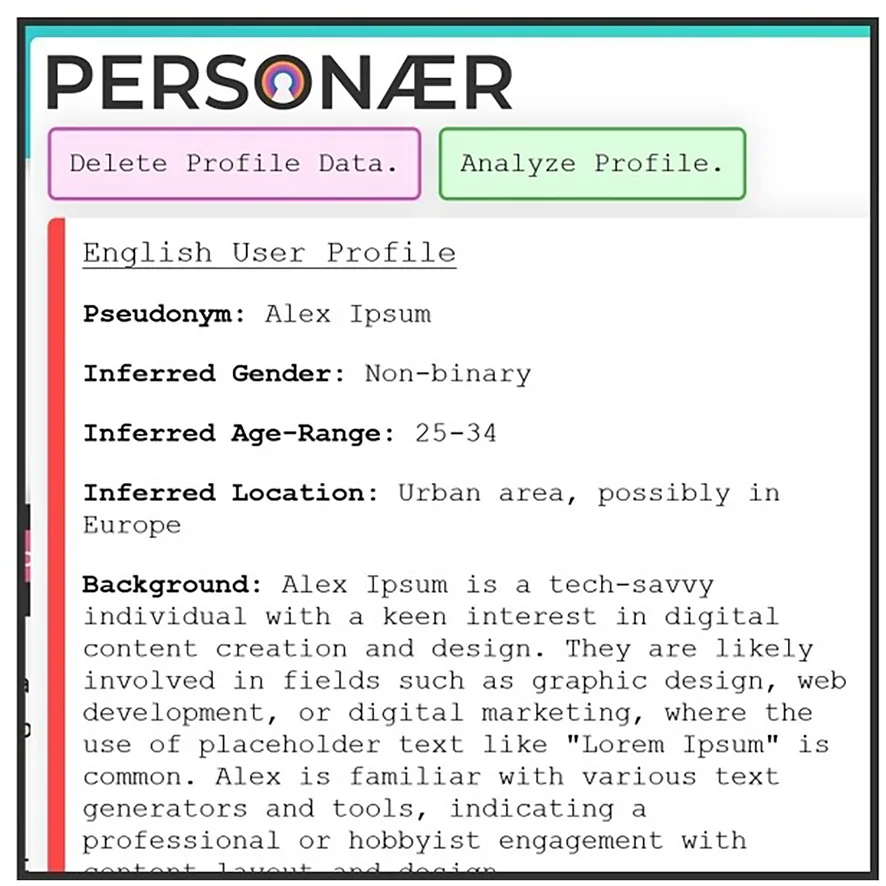
The PERSONÆR browser extension interface. Translates passive digital footprints gathered through keyword extraction into an inferred, narrative user persona. By structuring data into tangible fields like age, gender, and background narrative, the tool aims to bridge the bounded rationality gap and make the abstract risks of Algorithmic Profiling easier to understand (Kronhardt et al., 2024a).

The design of *PERSONÆR* directly addresses the Privacy Paradox of Algorithmic Profiling. While traditional ex-post TETs attempt to mitigate the lackluster protective behavior by visualizing disclosed data, they often rely on abstract representations or specific data types (like geolocation) that users struggle to connect to their personal identity (Murmann and Fischer-Hübner, 2017). By employing LLMs, which have been shown to effectively alter user perceptions on complex issues (Costello et al., 2024; Albers and Hassenzahl, 2024), *PERSONÆR* bridges this gap. By converting passive digital footprints—which can reveal intimate emotions and behaviors (Goda et al., 2015; Insel, 2017)—into a relatable narrative, the tool aims to make the abstract risks of Algorithmic Profiling tangible, helping users understand the implications of disclosing personal data (Hansen, 2008).

#### 3.1.3 Information Disorder and FallacyCheck

Information Disorder describes the pollution of the information ecosystem through three distinct categories: misinformation (false information shared without harmful intent), disinformation (false information deliberately created to harm), and malinformation (genuine information shared in a deliberately harmful way) (Wardle and Derakhshan, 2017; Scheibenzuber et al., 2021).

Information Disorder permeates digital ecosystems by leveraging specific cognitive deficits and biases. A primary driver is a lack of analytical reasoning, described in research as “laziness,” where users fail to critically evaluate content accuracy before sharing (Pennycook and Rand, 2019). This is compounded by a human bias toward novelty, which causes false information to spread more rapidly than the truth (Vosoughi et al., 2018).

Content, such as news or social media, within this sphere often utilizes logical fallacies to manipulate users into beliefs that are unsubstantiated or lack evidence (Jin et al., 2022). These manipulations are particularly effective in “echo chambers,” where pre-existing beliefs and ideologies attenuate the effectiveness of corrective measures (Walter et al., 2019; Zollo et al., 2017). Traditional mitigation strategies like *post-hoc* debunking often fail because they can trigger defensive responses, reinforcing the very misconceptions they aim to correct (Fernandez and Alani, 2018). The structural nature of Information Disorder shifts the burden onto the user to actively discern truth from fabrication in an environment designed to overwhelm their critical faculties.

To assist users in navigating this polluted ecosystem without triggering defensive responses, we designed *FallacyCheck*. *FallacyCheck* is a proactive browser extension that integrates directly into the user's reading environment to facilitate the critical assessment of news articles (Kronhardt et al., 2025b). The system operates in the background, extracting text from news articles to identify thirteen specific types of logical fallacies—such as “Ad Hominem,” “False Causality,” or “Appeal to Emotion” (Jin et al., 2022). Utilizing an LLM, the tool avoids binary truth labeling in favor of highlighting specific fallacious arguments within the article (see Figure 3). Upon hovering over a highlight, the user is presented with a contextual tooltip that defines the error and poses a non-leading question. For instance, rather than simply flagging a statement, the tool might ask the user, “Is it accurate to say that [Event A] is directly caused by [Event B]?” to prompt an immediate, user-driven verification of the claim's logic.

**Figure 3:**
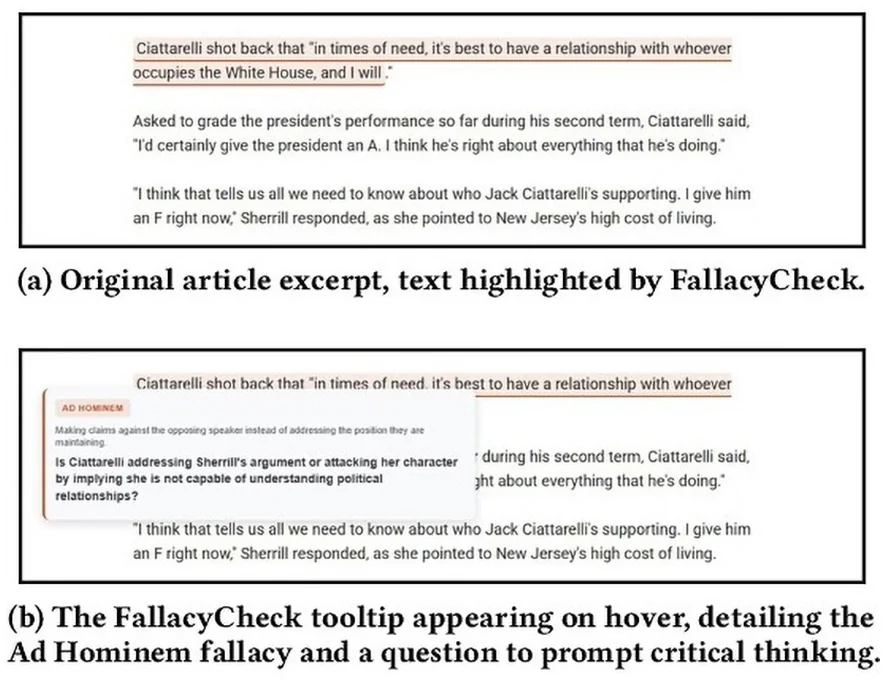
The FallacyCheck extension integrating Socratic nudging into the reading environment. **(a)** An excerpt from the Fox News Article “Final faceoff: Democrat, Republican nominees in key race for governor blast each other on debate stage” (https://www.foxnews.com/politics/final-faceoff-democrat-republican-nominees-key-race-governor-debate-final-time, last accessed 09.10.2025) with text highlighted by the tool to indicate a potential logical fallacy. **(b)** The contextual tooltip appearing on hover. The tool details the identified “Ad Hominem” fallacy and poses a non-leading question. This counteractive strategy disrupts passive consumption and prompts meta-cognitive reflection to combat analytical laziness (Kronhardt et al., 2025b).

*FallacyCheck* targets the user's cognitive processes by shifting focus from content verification to structural analysis, effectively operationalizing inoculation theory (McGuire and Papageorgis, 1961; McGuire, 1961) to build resistance against persuasive tactics. To counter the analytical laziness (Pennycook and Rand, 2019) that often facilitates the spread of misinformation, the system employs a nudging strategy (Horne et al., 2019; Pennycook et al., 2021) that actively interrupts passive consumption. The core mechanism—non-leading questioning—serves as a persuasive trigger (Fogg, 2009), forcing the user to engage in meta-cognitive reflection rather than defensive processing. By facilitating this self-generated refutation, the tool aims to avoid the “backfire effects” associated with direct corrections (Fernandez and Alani, 2018; Walter et al., 2019) and to foster a sustainable habit of critical inquiry.

### 3.2 Annotations

Having now described the mitigation tools and their corresponding phenomena in detail, we annotated them to consolidate overarching patterns into intermediate-level knowledge (see Figure 4). We categorize these annotations by how the systems influence user behavior, impact autonomy, and the modes through which the interventions operate.

**Figure 4:**
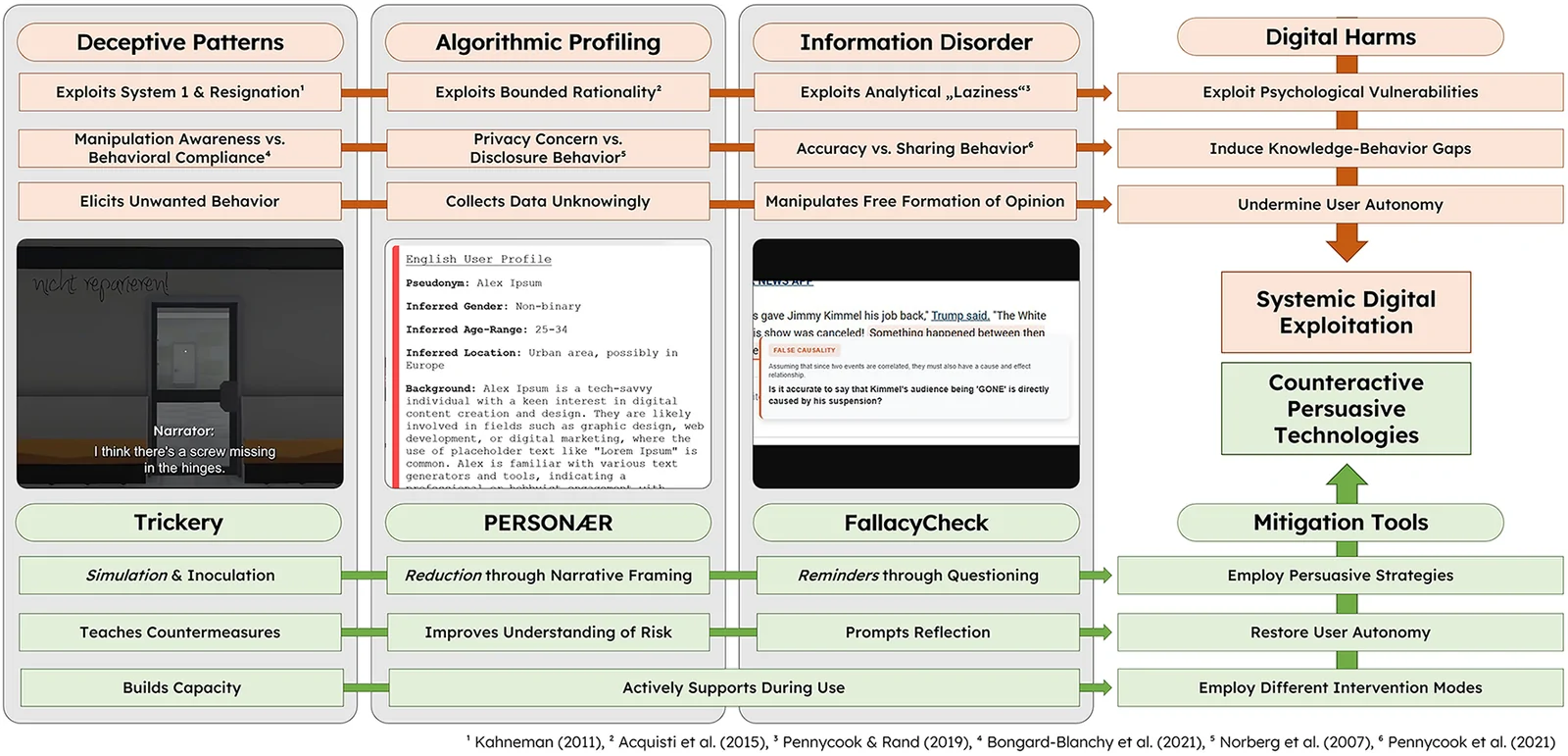
Annotated portfolio mapping the structural similarities across three distinct digital harms and their respective mitigation tools. The top section (orange) illustrates how Deceptive Patterns, Algorithmic Profiling, and Information Disorder individually operate to collectively exploit psychological vulnerabilities, exhibit Knowledge-Behavior Gaps, and undermine user autonomy. The bottom section (green) details how the corresponding mitigation tools (Trickery, PERSONÆR, and FallacyCheck) utilize specific persuasive strategies and intervention modes to counteract these exploits and restore user autonomy (Kronhardt et al., 2024b,a, 2025b).

#### 3.2.1 Influencing user behavior

Annotations across the three domains point toward recurring structural patterns in how digital harms operate and how they can be effectively countered.

- **Digital Harms Exploit Psychological Vulnerabilities:** The digital harms we annotated do not simply overwhelm users with information; they actively target specific cognitive shortcuts. Deceptive Patterns capitalize on System 1 resignation, bypassing rational thought to secure compliance. Algorithmic Profiling exploits bounded rationality, banking on a user's inability to weigh abstract, long-term privacy risks against immediate digital gratification. Information Disorder leverages analytical “laziness,” where the human bias toward novelty overrides the critical evaluation of content.
- **Digital Harms Induce Knowledge-Behavior Gaps:** The exploitation of these vulnerabilities in the digital harms we annotated results in a pronounced rift between a user's reflective knowledge and their actual in-the-moment behavior. Across the portfolio, this manifests as clear behavioral contradictions documented in the prior literature on each phenomenon: users are often aware of manipulation through deceptive patterns but comply with the manipulative platform anyway (Manipulation Awareness vs. Behavioral Compliance) (Bongard-Blanchy et al., 2021; Di Geronimo et al., 2020); they state strong privacy concerns but readily disclose personal data (Privacy Concern vs. Disclosure Behavior) (Norberg et al., 2007; Taddicken, 2014); and they value truth and know they should be checking information they consume for accuracy but actively share unverified, fallacious news (Accuracy vs. Sharing Behavior) (Pennycook et al., 2021). We emphasize that these contradictions are properties of the digital harms themselves, established in prior work, rather than measurements obtained from our own three tools, whose individual evaluations each targeted narrower, tool-specific questions. Critically, each contradiction demonstrates that reflective knowledge alone is insufficient to produce correspondingly aligned behavior; it is precisely this insufficiency that motivates a proactive, rather than purely informational, response.
- **Mitigation Tools Employ Persuasive Strategies:** Rather than merely presenting passive warnings, our mitigation tools actively utilize persuasive strategies (Oinas-Kukkonen and Harjumaa, 2009) to disrupt these behavioral gaps. Each tool operationalizes its high-level design strategy by grounding it in a specific underlying cognitive or functional mechanism that defines how the intervention is structured. In *Trickery*, the persuasive strategy of *Simulation* is motivated through *Inoculation Theory*, exposing users to weakened forms of deception to teach practical countermeasures. In *PERSONÆR*, the strategy of *Reduction* is executed through the mechanism of *Narrative Framing*, translating abstract tracking data into a tangible, relatable persona to improve the user's understanding of digital risk. In *FallacyCheck*, the strategy of contextual *Reminders* is operationalized through the mechanism of *Non-leading Questioning*, which directly interrupts passive consumption to prompt meta-cognitive reflection.

#### 3.2.2 Influencing user autonomy

The ultimate stakes of the annotated digital harms—and the interventions designed to counter them—are deeply tied to the user's self-determination.

- **Digital Harms Undermine User Autonomy:** When environments exploit psychological traits to enforce a Knowledge-Behavior Gap, they actively thwart the user's basic psychological need for autonomy. This decoupling strips the user of authentic choice, forcing compliance through external regulation. Deceptive patterns elicit unwanted behavior, profiling infrastructures quietly collect data without the user's explicit knowledge, and information disorder manipulates the free formation of opinion, effectively undermining user autonomy.
- **Mitigation Tools Restore User Autonomy:** To counteract this, the annotated mitigation tools purposefully deploy persuasion, not to impose a new, system-defined “good” behavior, but to actively dismantle the pre-existing environmental manipulation and restore user autonomy. By disrupting automaticity, these mitigation tools buy users the critical cognitive resources required to make self-determined choices, which were inhibited by the environmental manipulation. Their overarching goal is to shift the user away from forced external compliance and toward integrated regulation, allowing their digital actions to align with their actual, reflective values.

#### 3.2.3 Intervention modes

While the goal of restoring autonomy is shared across the tools, the portfolio suggests that interventions may need to be applied across different temporal contexts.

- **Mitigation Tools Employ Different Intervention Modes:** Interventions operate through distinct functional modes. *Trickery* focuses on building capacity, taking place outside the immediate context of vulnerability. It serves as a restorative training environment designed to build long-term internal resistance before the user encounters real-world harm. In contrast, *PERSONÆR* and *FallacyCheck* actively support during use. They provide real-time, situational scaffolding directly embedded within the user's browsing environment. They actively compensate for bounded rationality and analytical laziness precisely at the moment of potential exploitation.

We suggest these structural overlaps resonate with established psychological concepts, indicating that they may have utility beyond our specific portfolio. System 1 automaticity (Kahneman, 2011), bounded rationality (Acquisti et al., 2015), and analytical laziness (Pennycook and Rand, 2019) are well-established drivers of user vulnerability, documented across several distinct digital harms. Because these exploitative environments target shared cognitive bottlenecks, we infer that the interventions required to counteract them may converge on similar structural strategies—specifically, the deployment of active persuasion to disrupt automaticity and bridge the Knowledge-Behavior Gap. This provisional convergence motivates our attempt to abstract the shared mechanics of these specific design cases into intermediate-level knowledge, pending validation against a broader, independently developed corpus.

## 4 Synthesizing intermediate-level knowledge

Having identified these structural similarities in our annotated portfolio, we now move beyond viewing these digital harms and their respective mitigation tools as isolated phenomena. To maintain methodological clarity, it is important to explicitly delineate the scope of the following synthesis:

The preceding annotations serve as our grounded evidence, illustrating how our three specific mitigation tools attempt to counteract instances of digital harm by bridging a Knowledge-Behavior Gap. From this bounded portfolio, we make a provisional theoretical inference: because Deceptive Patterns, Algorithmic Profiling, and Information Disorder appear to exhibit recurring structural similarities in both how they operate and how they can be countered within our portfolio, we infer the potential for recurring structural commonalities across distinct phenomena of digital harm beyond our portfolio.

Consequently, we build upon this inference to hypothesize two novel constructs of intermediate-level knowledge. Rather than proposing established, universal laws of human-computer interaction, we offer these constructs as provisional tools for theoretical sense-making and applied design, to be tested and validated by the research community across broader digital ecosystems. Drawing upon RtD epistemology, this synthesis proposes:

- **Systemic Digital Exploitation (SDE)** as a *conceptual construct* *(Stolterman and Wiberg, 2010)* that brings together disparate instances of digital harm into a unified theoretical understanding of how hostile environments undermine user autonomy.
- **Counteractive Persuasive Technologies (CPT)** as a *bridging concept* *(Dalsgaard and Dindler, 2014)* that connects concrete, restorative design practices to the macro-psychological principles of Self-Determination Theory, facilitating exchange between overarching theory and practice.

This section proposes the two constructs and establishes their status as intermediate-level knowledge; it does not delimit them. Section 5 then develops both: we outline the boundary conditions that bound SDE and test each against a phenomenon that fails it alone, and present the CPT design process that adapts established persuasive design models for adversarial contexts and grounds the framework's ethical mandate.

### 4.1 Systemic Digital Exploitation as a conceptual construct

While Deceptive Patterns, Algorithmic Profiling, and Information Disorder operate through distinct modalities, the annotated portfolio of our applied research points toward a shared underlying logic. These phenomena function by actively exploiting psychological vulnerabilities to inhibit the application of existing knowledge during decision-making. To capture this structural similarity, we propose the following provisional definitions:

**Systemic Digital Exploitation (SDE):** The strategic design of digital environments to actively exploit human psychological vulnerabilities to bypass rational decision-making, induce a *Knowledge-Behavior Gap*, and thwart user autonomy.

**Knowledge-Behavior Gap:** The central mechanism leveraged by SDE, defined as a structurally induced rift wherein a user's in-the-moment actions are deliberately decoupled from their reflective knowledge and long-term values.

Rather than proposing SDE as a definitive, universal psychological theory, we explicitly position it as a *conceptual construct* (Stolterman and Wiberg, 2010). As established in Subsection 2.4, a conceptual construct serves as an intellectual or logical construction that occupies the midway point between isolated concepts and all-encompassing grand theories (Stolterman and Wiberg, 2010). SDE acts as a conceptual construct by abstracting specific instances of digital harm into a generalized conceptual vocabulary to help advance HCI theory. This allows researchers to analyze overarching structural manipulations across digital ecosystems and focus on theoretical advancement rather than merely cataloging isolated occurrences.

By targeting vulnerabilities—such as the reliance on System 1 processing, bounded rationality, or analytical laziness—SDE encourages behaviors (e.g., forced consent, data disclosure, misinformation sharing) that contradict a user's own knowledge or long-term interests. Viewed through the lens of SDT, this decoupling is an active thwarting of the user's basic psychological need for autonomy (Ryan and Deci, 2000). SDE leverages the environment to force compliance (external regulation), stripping the user of authentic, integrated choice. The definition alone does not delimit the construct: it states what SDE is, but not what falls outside it. Four provisional boundary conditions that perform that delimitation, and the counter-examples establishing that each can fail independently of the others, follow in Subsection 5.1.

### 4.2 Counteractive Persuasive Technologies as a bridging concept

Given that SDE actively thwarts user autonomy by means of a Knowledge-Behavior Gap, we argue that simply providing users with more information may be insufficient to combat it. Doing so may increase the user's amount of knowledge but does not target the persuasive nature of SDE. Based on our annotated portfolio, we hypothesize that interventions may need to actively employ counter-persuasive strategies to restore user autonomy, thereby supporting the user's right to mental self-determination. To capture this class of interventions, we propose the following provisional definition:

**Counteractive Persuasive Technologies (CPT):** Digital interventions that actively deploy persuasive strategies to counteract Systemic Digital Exploitation, bridging the Knowledge-Behavior Gap to restore user autonomy and support mental self-determination.

To operationalize this definition and structure the design of CPTs, we extract four core components directly from the structural similarities observed in our annotations. Every CPT intervention can be mapped across these elements:

1. **Digital Exploitation Phenomenon:** The specific adversarial SDE mechanism embedded in the environment (e.g., Deceptive Patterns).
2. **Exploited Psychological Mechanism:** The cognitive vulnerability targeted to create the Knowledge-Behavior Gap (e.g., System 1 Resignation).
3. **Intervention Mode:** The functional temporal context of the tool, categorized as either *Capacity-Building* (restorative training outside the vulnerable context) or *Scaffolding* (real-time compensatory support during active use).
4. **Counteractive Persuasive Strategy:** The specific persuasive method deployed to restore autonomy (e.g., Inoculation, Narrative Framing, Socratic Nudging).

By formalizing these four components, we position CPT as a *bridging concept* (Dalsgaard and Dindler, 2014)—a form of intermediate-level knowledge that facilitates a bidirectional exchange between abstract overarching theory and concrete design practice (see Subsection 2.4). A summary mapping of these four components across our three exemplar interventions can be found in Table 1. Our formulation of CPT fulfills the three defining constituents of a bridging concept:

**Table 1:** Application of the CPT framework.

Tool	Digital exploitation phenomenon	Exploited psychological mechanism	Intervention mode	Counteractive persuasive strategy
Trickery	**Deceptive patterns** *Gap is Awareness-Compliance* (Bongard-Blanchy et al., 2021; Di Geronimo et al., 2020)	**System 1 & resignation** Exploits automaticity and learned helplessness to secure forced consent. (Kahneman, 2011)	Capacity-building	**Inoculation** Simulates “Gamified Deceptive Patterns” to force a shift from *Automaticity*→*Analysis*, building internal resistance.
PERSONÆR	**Algorithmic profiling** *Gap is concern-disclosure* (Norberg et al., 2007; Taddicken, 2014)	**Bounded rationality** Exploits the inability to weigh long-term abstract risks against immediate gratification. (Acquisti et al., 2015)	Scaffolding	**Narrative framing** Uses LLMs to transform data into stories, moving from *Abstraction*→*Affect* to make privacy risks tangible.
FallacyCheck	**Information disorder** *Gap is accuracy-sharing* (Pennycook et al., 2021)	**Analytical laziness** Exploits the failure to engage critical faculties during passive consumption. (Pennycook and Rand, 2019)	Scaffolding	**Socratic nudging** Interrupts reading with non-leading questions to shift from *Passivity*→*Scrutiny* via self-reflection.

This summary maps the identified Digital Exploitation Phenomena and their Exploited Mechanisms to the specific CPT Interventions developed to counteract them. It details how each tool employs a specific Mode and Strategy to bridge the Knowledge-Behavior Gap. Each Knowledge-Behavior Gap and Exploited Psychological Mechanism is cited to the prior literature establishing it for the corresponding phenomenon; see also Subsection 3.2, where the same evidence is discussed in full.

#### 4.2.1 Theoretical grounding

As highlighted in our *Influencing User Autonomy* annotation, tools designed to combat SDE cannot remain neutral. CPT is firmly anchored in SDT and the traditional domain of PT. While traditional PTs often nudge users toward system-defined behavioral targets, CPTs utilize persuasion strictly to disrupt pre-existing environmental manipulation.

To prevent CPTs from devolving into a new form of soft paternalism, we establish a strict ethical criterion: *A CPT must not mandate a specific behavioral outcome (e.g., forcing a user to reject data collection), but must solely optimize for the restoration of reflective decision-making*. Drawing on SDT's Basic Psychological Needs Theory and Organismic Integration Theory (Ryan and Deci, 2000), the theoretical goal of a CPT is to buy the user the cognitive resources necessary to move away from forced compliance (external regulation) and toward choices that align with their authentic values (integrated regulation)—even if that autonomously formed choice ultimately entails accepting a digital risk. We state this criterion here as a constituent of the bridging concept; the argument for why it holds, and the design process that enforces it, follow in Section 5.2.

#### 4.2.2 Design articulations

Bridging concepts must provide parameters that designers can manipulate (Dalsgaard and Dindler, 2014). For CPT, the latter two components of our framework serve as these exact articulations. They define how the intervention actively bridges the Knowledge-Behavior Gap. The *Intervention Mode* gives designers a functional target, while the *Counteractive Persuasive Strategy* provides the concrete method to neutralize the exploited psychological mechanism and restore analytical processing.

#### 4.2.3 Exemplars

Finally, a bridging concept must be accountable to practical exemplars that demonstrate the scope of its application (Dalsgaard and Dindler, 2014). Our annotated portfolio provides these direct foundations: *Trickery* (Kronhardt et al., 2024b) exemplifies Capacity-Building by utilizing inoculation and simulation to combat System 1 automaticity. *PERSONÆR* (Kronhardt et al., 2024a) operates as Scaffolding, utilizing narrative framing to make abstract data tangible and combat bounded rationality. *FallacyCheck* (Kronhardt et al., 2025b) acts as Scaffolding, employing Socratic nudging to disrupt the passive consumption associated with Information Disorder.

By synthesizing our portfolio through this bridging concept, we provide a structured vocabulary for the HCI community to intentionally design systems that actively defend mental self-determination against SDE.

## 5 Discussion

In retrospectively analyzing our applied research, we observe that these isolated phenomena of digital harm and their corresponding mitigation tools can be understood through a shared architectural lens. By comparing the psychological vulnerabilities exploited by the digital harms in our annotated portfolio, our synthesis suggests they share a core mechanism, which we conceptualize as SDE. Likewise, observing the structural similarities in how the tools in our annotated portfolio mitigate harm led us to outline CPT as a viable mitigative approach. To further develop this perspective, we outline the initial boundary conditions of SDE and then present the CPT design process, which adapts established persuasive design models for adversarial contexts and grounds the framework's ethical mandate.

### 5.1 Boundary conditions of Systemic Digital Exploitation

The name Systemic Digital Exploitation was deliberately chosen to reflect the conditions which bound the construct. Each of the three terms (Systemic, Digital, and Exploitation) performs analytical work, and each must therefore be independently satisfiable and independently falsifiable. To these we add a fourth condition concerning the distribution of harm, which the name does not carry but which is required to separate exploitation from disclosed and mutually beneficial exchange. To distinguish SDE from adjacent harms, we propose that a phenomenon must meet all four criteria to fall under this definition:

- **Systemic (vs. Isolated Attacks):** The mechanism must be an external, sophisticated barrier embedded directly into digital platforms. It represents a pervasive feature of the broader digital ecosystem. The operative test is not one of scale or platform count, but of address: a mechanism is systemic when encountering it forms part of the ordinary, expected experience of a platform's general user base. It is not systemic when it is directed at a particular user or at a specifically selected subset of users.
- **Digital (vs. Physical Environments):** The manipulation must occur within digital ecosystems and interfaces. While physical environments routinely employ persuasive tactics, SDE is confined to digital platforms, where the user's access to the choice environment is itself furnished by the party arranging it. A physical arrangement may be weighted toward one option, but it is perceived directly, every option within it is present to the person standing before it, and inspecting it is a capacity of their own. A digital environment is present to the user only as it is rendered, and an option that is not rendered is not merely obscured but absent, leaving nothing to inspect and no vantage point from which the arrangement could be checked against the thing arranged. The operative test is therefore whether the environment supplies the user's entire evidential basis for the decision it presents.
- **Exploitation of Psychology (vs. Capability Constraints):** The digital environment acts as a disabling barrier by targeting and exploiting inherent human psychological traits. Specifically, these systems manipulate users by capitalizing on cognitive vulnerabilities, such as the human reliance on efficient, automatic processing (System 1) over effortful reflection (System 2), thereby exploiting a Knowledge-Behavior Gap. This psychological manipulation fundamentally distinguishes the phenomenon from constraints that operate on a user's capability rather than on their cognition. Where a barrier removes or restricts what a user is able to do, their knowledge and their behavior remain coupled, however adverse the constraint: they know what they would prefer, and they are simply prevented from enacting it. No Knowledge-Behavior Gap arises, because no gap is possible where no alternative action is available. SDE, in contrast, leaves the user's capability intact and instead targets the decision itself; it relies on the speed and automaticity of the exploited situation to quietly decouple a user's reflective knowledge from their actual behavior, prompting quick decisions without the user immediately realizing the manipulation has occurred. The diagnostic question is therefore not whether the user was adversely affected, nor whether they were formally informed, but whether the mechanism operated by foreclosing an action or by corrupting the choice between actions that remained open.
- **Asymmetric Harm (vs. Balanced Exchange):** The mechanism must distribute costs and benefits asymmetrically against the user, and must do so without a transparently disclosed proposition that the user could evaluate on its stated terms. We deliberately do not specify an absolute threshold of harm, since what counts as an acceptable cost is precisely the judgment that belongs to the user, and precisely the judgment that SDE removes from them. The criterion is therefore relative and procedural rather than absolute: it asks whether the exchange was disclosed and evaluable, not whether it was favorable.

These four conditions are jointly necessary. Deceptive Patterns, Algorithmic Profiling and Information Disorder each satisfy all four, which is what permits their treatment under a shared construct. The conditions do analytical work, however, only insofar as each can fail independently of the others. We therefore work through four illustrative phenomena, each of which satisfies three conditions and fails exactly one, applying every condition in turn rather than asserting the verdict (see Table 2):

- *Failing Systemic*: Targeted Social Engineering Attack. Consider an attacker who researches a particular employee, impersonates a known colleague or supplier, and contacts them directly to obtain a credential or authorize a payment. The mechanism is plainly *Digital*, executed through messaging and spoofed interfaces that the attacker adapts to the individual recipient. It *Exploits Psychology* in the fullest sense, manufacturing urgency and impersonating authority to secure action before reflection intervenes; the Knowledge-Behavior Gap is precisely the point, since victims typically know that such approaches exist and act against that knowledge anyway because of how the approach is structured. Its harm is *Asymmetric* and undisclosed, the attacker gaining entirely at the victim's expense. It fails only on *Systemic*. Applying the test of address, being approached in this way is not part of the ordinary, expected experience of a platform's general user base, but the consequence of having been selected, whether individually or as a member of a harvested list. The mechanism is an event that befalls particular people, not a standing condition of the environment those people inhabit. This matters for mitigation: a countermeasure can be aimed at the attack and at the infrastructure behind it, whereas SDE affords no such target, because there is no discrete attack to intercept.
- *Failing Digital*: Supermarket Product Placement. Supermarkets often place expensive product variants at eye-level, while their cheaper alternative sits low in the shelf. The practice is *Systemic*, since encountering it is the ordinary and expected experience of every shopper in the store rather than something directed at particular customers. It *Exploits Psychology*, capitalizing on visual salience and the reliance on automatic processing during routine, low-attention decisions, and the resulting behavior departs from what shoppers would report valuing if asked. Its harm is mildly *Asymmetric*, the placement fee being paid by the brand and recovered from the shopper who did not choose the cheaper shelf. It fails only on *Digital*. The store arranges the shelf, but it does not furnish the shopper's perception of it. Every variant the store stocks is present to the person standing in the aisle, the cheaper option among them, and the countermeasure of looking at the lower shelf is a capacity the shopper brings rather than an accordance the store grants. The placement can therefore weight the decision but cannot constitute it: the arrangement remains checkable against the thing arranged with minimal effort, and a shopper who suspects the eye-level product is the more expensive one can establish as much on the spot. This is what the digital case withholds, where an option the interface does not render or hides behind additional steps is not present to be found.
- *Failing Exploitation of Psychology*: Firmware Updates that Disable Third-Party Consumables. Printer manufacturers have distributed firmware updates that cause already-purchased devices to reject third-party or refilled ink cartridges which the printer had previously accepted.^1^ The practice is *Systemic*, applying to every owner of an affected model as a standing condition of the product rather than to selected individuals. It is unambiguously *Digital*, enacted through software distributed to devices already in users' possession and capable of altering their behavior after purchase. Its harm is *Asymmetric* and undisclosed: the owner's hardware and remaining stock of consumables lose value through no act of their own, the benefit accrues entirely to the manufacturer's aftermarket, and the change is imposed after the purchase decision has been made and can no longer be revisited. It fails only on *Exploitation of Psychology*. The update operates on capability rather than cognition. It forecloses an action outright rather than corrupting a choice among actions that remain open, and the owner confronted with a rejected cartridge wants precisely what they can no longer do. Their reflective knowledge and their behavior never come apart, because no behavioral alternative exists to be decoupled from. The case shows that the exploitation condition is not a measure of how adversely a user is treated, but of the vector through which the treatment operates: a manufacturer may act against a user's interests comprehensively and still not exploit them in the sense SDE requires.
- *Failing Asymmetric Harm*: Retail Loyalty Stamp Cards. The practice is *Systemic* across retail and e-commerce, encountered as an ordinary feature of the sector rather than offered to selected customers, and its contemporary implementations are *Digital*, administered through applications that track purchases and sit within environments that could vary their terms per customer. It also, and importantly, *Exploits Psychology*: the goal-gradient effect accelerates purchasing as the reward nears (Kivetz et al., 2006), artificial initial progress inflates commitment (Nunes and Drèze, 2006), and loss aversion (Kahneman and Tversky, 1979) over accumulated stamps deters switching to a competitor. The card is therefore not a neutral instrument, and a customer may well buy a tenth coffee they did not want in order to complete a row. It fails, nonetheless, on *Asymmetric Harm*. The proposition is stated on the face of the card: concentrate your custom here, and receive a discount or a free product. Its terms are legible before the first stamp, the exchange may be declined without cost, and a customer who judges the bargain poor can simply leave the card unused. The imbalance remains available to the user's own evaluation. This is the case we regard as most instructive, because it isolates the harm condition from the exploitation condition entirely: a mechanism may make use of a genuine psychological vulnerability and still fall outside SDE, provided the exchange it proposes is disclosed and the user's capacity to judge it survives intact.

**Table 2:** Boundary conditions of systemic digital exploitation.

Phenomenon	Systemic	Digital	Exploits psychology	Asymmetric harm
Deceptive patterns	✓	✓	✓	✓
Algorithmic profiling	✓	✓	✓	✓
Information disorder	✓	✓	✓	✓
Targeted social engineering attack	*Fails:* Addresses selected individuals, not a standing property of the environment.	✓	✓	✓
Supermarket product placement	✓	*Fails:* Shopper perceives the shelf directly; all options checkable on the spot.	✓	✓
Firmware updates disable third-party consumables	✓	✓	*Fails:* Operates on capability, not cognition; no alternative action to decouple from.	✓
Retail loyalty stamp card	✓	✓	✓	*Fails:* Exchange disclosed on its face and declinable; user's judgement remains intact.

This summary tests the four proposed conditions against phenomena that satisfy three conditions and fail exactly one, demonstrating that each condition is independently falsifiable rather than a restatement of the construct. The three phenomena of our annotated portfolio satisfy all four conditions simultaneously.

Taken together, these cases mark out the construct from four directions. The phishing case shows that adversarial intent is not sufficient for SDE, since a mechanism aimed at selected people can be intercepted as the discrete event it is; the supermarket case shows that a weighted choice architecture is not sufficient either, since a shopper who perceives the shelf directly retains the means to check it. The firmware case shows that acting against a user's interests is not sufficient, and the loyalty card that exploiting a psychological vulnerability is not sufficient. Neither adversity, nor influence, nor harm alone identifies the phenomenon we mean to name. What SDE picks out is narrower and, we would argue, more troubling than any of them: a standing condition of the environment, furnishing the user's own view of the decision, which leaves their capabilities intact and their knowledge unchallenged and then arranges the moment of decision so that neither can be brought to bear. The user is not prevented from acting on their values, nor argued out of them. They are routed past them, with no discrete attack to intercept and no vantage point from which the arrangement could be seen for what it is. It is this specific decoupling, rather than harm or influence as such, that the four conditions are designed to isolate, and that the interventions we turn to next are designed to reverse.

### 5.2 Designing Counteractive Persuasive Technologies

The introduction of CPTs inherently raises a critical ethical paradox: if CPTs utilize the very same persuasive mechanisms as the systems they aim to counteract, do they not risk perpetuating the same harms? Echoing foundational debates within the HCI community regarding the ethical boundaries of PT (Rahman and Adaji, 2024), “fighting persuasion with persuasion” walks a precarious line. If a designer attempts to “save” users from digital harms by coercing them into a predetermined “safe” or “correct” behavior, they inadvertently fall into the trap of soft paternalism, replacing one form of external regulation with another (Purpura et al., 2011; Smids, 2012; Atkinson, 2006).

Our answer begins definitionally: a CPT is restorative by construction, since an intervention that prescribes a behavioral outcome of its own is not a CPT at all (per Section 4). That definition, however, does not enforce itself. A designer working in good faith can still drift from counteracting an exploitation toward correcting a behavior, and nothing in the definition alone would catch the drift. Upholding the restorative mandate therefore requires a design process disciplined enough to keep an intervention tethered to it: one that obliges the designer to identify the exploitation being countered before designing the countermeasure, so that the intervention's persuasive force has a fixed and external referent rather than a goal of the designer's choosing. This is why the CPT framework does not simply inherit established persuasive design methods but adapts them. We therefore present the adaptation first, and return to the ethical account once the process that supports it is in place.

#### 5.2.1 Recontextualizing persuasive design for adversarial environments

CPTs operate as a specialized subgroup within the broader domain of PTs. While CPTs employ the strategies of established frameworks like PSD (Oinas-Kukkonen and Harjumaa, 2009) or FBM (Fogg, 2009), they diverge in their functional intent and operational context.

As established in Subsection 2.1, traditional PTs often prioritize system-defined compliance, leveraging System 1 automaticity and external regulation in ways that risk overriding user agency through soft paternalism (Purpura et al., 2011; Smids, 2012), even if their goals are benevolent. In contrast, CPTs do not impose a predefined “good” behavioral goal. Rather, we conceptualize them to serve as defensive interventions in adversarial environments. By proactively disrupting pre-existing environmental manipulation, CPTs aim to support the restoration of users' autonomy and right to mental self-determination. This buys users the cognitive resources necessary to move away from the forced compliance of the adversarial environment and toward integrated regulation, ultimately allowing them to align their actions with their own authentic values.

This divergence in intent is what the adapted process operationalizes, and it is where CPT contributes something PSD does not supply. PSD's contextual analysis asks the designer to characterize the persuasive intent, on the assumption that the party exerting influence on the interaction is either the system creators, the distributors, or the users themselves. In an adversarial environment that assumption fails: influence is already being exerted before the designer arrives, and a framework that does not require this to be identified cannot distinguish a countermeasure from an additional influence. The CPT framework therefore builds directly upon the established two-step process of the PSD model—*Analyzing the Persuasion Context* and *Design of System Qualities* (Oinas-Kukkonen and Harjumaa, 2009)—but recontextualizes both phases accordingly (see Figure 5). Rather than analyzing how to persuade a user toward a system-defined behavioral goal, practitioners systematically design agency-restoring interventions by moving through two adapted phases.

**Figure 5:**
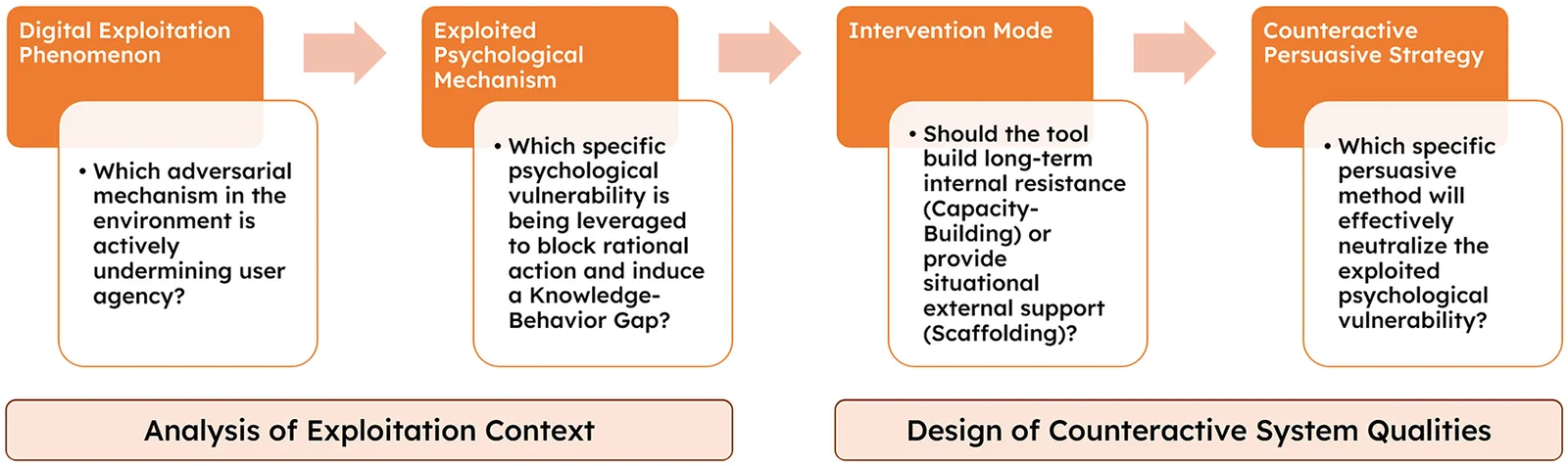
The Counteractive Persuasive Technology (CPT) design process. The framework outlines four sequential, theoretically-grounded questions to guide designers in systematically auditing adversarial environments and formulating autonomy-restoring interventions.

#### 5.2.2 Step 1: analysis of *exploitation* context

Designers operating in hostile environments must first identify and name the specific Digital Exploitation Phenomenon *already* embedded within the system architecture, together with the psychological mechanism it exploits. This identification is a necessary precondition for any CPT: an intervention that cannot name a pre-existing SDE to counteract is not a CPT, since it would then constitute an independent behavioral influence rather than a countermeasure. This adapts PSD's traditional contextual analysis by requiring designers to deconstruct the SDE through two inquiries:

- **Digital Exploitation Phenomenon:** Designers identify the adversarial mechanism (e.g., Deceptive Patterns) embedded in the system by asking: *Which adversarial mechanism in the environment is actively undermining user agency?*
- **Exploited Psychological Mechanism:** To understand how this exploitation functions, designers must pinpoint the precise cognitive vector by asking: *Which specific psychological vulnerability is being leveraged to block rational action and induce a Knowledge-Behavior Gap?*

#### 5.2.3 Step 2: design of *counteractive* system qualities

Once the adversarial context is mapped, designers advance to the second PSD phase: designing the actual system qualities to counteract it. Here, CPT integrates FBM and SDT to ensure interventions are functionally viable and psychologically supportive:

- **Intervention Mode (Informed by FBM):** Designers operationalize FBM's concept of *Ability* by evaluating the user's *least available resource* at the moment of exploitation (e.g., time, attention, or cognitive load). Based on this constraint, designers ask: *Should the tool build long-term internal resistance (Capacity-Building) or provide situational external support (Scaffolding)?* Because the scarcest resource is a property of the individual user in a particular moment rather than of the exploitation in the abstract, this question does not resolve into a single fixed answer for all users. A Scaffolding intervention calibrated to a user with little attention to spare may be intrusive to a user with more, and a scaffold that cannot be adjusted implicitly assumes a uniform resource profile that FBM itself does not support. Designers should therefore treat the *intensity* of a Scaffolding intervention as a design parameter alongside its mode, and, where the deployment context permits, place that parameter under the user's control. The same reasoning extends to deployment across sectors and user groups, where the scarcest resource differs systematically rather than incidentally.
- **Counteractive Persuasive Strategy (Informed by SDT):** Synthesizing the adversarial intent and the user's constrained resources, designers ask: *Which specific persuasive method will effectively neutralize the exploited psychological vulnerability?* Drawing upon SDT, the selected strategies must actively move the user away from the external compliance caused by the SDE and toward *integrated regulation*. The counteractive persuasive strategy should satisfy basic psychological needs to help the user internalize their own authentic values against the adversarial prompt.

#### 5.2.4 How the design process answers the paternalism objection

With the process in place, we can return to the paradox with which this section opened. Our first response is definitional: *a system that forces a predefined “good” behavioral outcome is, by definition, not a CPT*. While prior research frequently critiques traditional PTs for their paternalistic tendencies, the CPT design process avoids this by explicitly rejecting system-defined behavioral targets. Its sole ethical mandate is restorative: the defense of the user's fundamental right to mental self-determination (Faraoni, 2023) and support of their basic psychological need for autonomy (Ryan and Deci, 2000). The final behavioral decision remains with the user.

This property, however, does not rest on definition alone. We acknowledge the counterargument that any persuasive strategy, including Socratic nudging or narrative framing, shapes the decision space, and that friction is itself a design choice carrying directional intent. Our response is that in the contexts CPTs address—phenomena of SDE —the relevant comparison is not between a neutral decision space and a biased one. The user's decision space is *already* biased by the SDE, systematically and often without their awareness, and they presently have no structural guardrail against it. The question is therefore not whether a CPT exerts directional force, but in which direction and under what constraint.

Here the design process is decisive, and its two inquiries do distinct work. As established in Section 5.2.2, a CPT may only be constituted in response to a named, pre-existing *Digital Exploitation Phenomenon*. This fixes the *referent* of the intervention's persuasive force: it is aimed at reversing an identified, externally imposed bias rather than at an outcome of the designer's choosing, which is what distinguishes friction that is *corrective* of a documented exploitation from friction that is *directive* toward a designer's goal. Isolating the exact *Exploited Psychological Mechanism* then fixes the intervention's *scope*, forcing an exploration of precisely what must be neutralized and preventing mission creep: the persuasion is deployed solely to bridge the targeted Knowledge-Behavior Gap, not to dictate the user's final choice. The definitional claim and these procedural requirements are therefore mutually dependent: neither alone would be sufficient, and it is the design process that renders the definition enforceable.

#### 5.2.5 Illustration and designer accountability

To illustrate, we can refer back to the PERSONÆR tool: The tool does not aim to stop data collection and algorithmic profiling—this would theoretically be achievable through a tool that simply blocks any known tracking mechanisms without any user interaction. Likewise, this could be achievable through directed persuasion via a tool that actively warns users not to visit sites known for tracking users extensively—a normative claim with an explicit behavioral goal. Instead, PERSONÆR offers the users a look at what their algorithmic profile might look like. The decision to change their behavior then lies entirely with the user. If they deem the profile harmless, they might approve of tracking for the sake of reaping the benefits of personalized content. If they deem the profile too personal or otherwise dangerous, they may reconsider consenting to tracking and may even exercise their rights to data deletion (e.g., through the GDPR). Either way, the decision was made with a reflexive understanding of what can be extrapolated from their online behavior, neutralizing the SDE of Algorithmic Profiling, which obscures this information, only presenting the users with the benefits (e.g., personalized content) over potential consequences.

We note, finally, that the framework constrains the designer's method but cannot guarantee the designer's fidelity to it. A practitioner may name an SDE phenomenon *pro forma* and proceed to build an intervention that quietly pursues an outcome of their own choosing, and no definitional boundary absolves the designer from the diligence required to prevent their own biases from slipping into the interface. What the CPT design process offers is not a technical impossibility of misuse, but an explicit and auditable standard against which an intervention can be assessed: the claim to be a CPT is falsifiable, since a tool that cannot point to the exploitation it counteracts, or that optimizes for a behavioral outcome rather than for restored deliberation, fails the definition on inspection. Responsibility for meeting that standard remains with the designer.

This design process exemplifies CPT's position as a *bridging concept* (Dalsgaard and Dindler, 2014). It provides both concrete design articulations *and* informs how overarching theoretical frameworks like PSD, FBM, and SDT are executed in practice. By translating abstract concepts of cognitive resource scarcity, integrated regulation, and adversarial intent into an actionable design process, CPT aims to bridge the gap between macro-level psychological theory and the applied engineering of autonomy-restoring systems right at the point of decision-making. Ultimately, grounded in SDT, the success metric of a CPT is not compliance, but the facilitation of integrated regulation. By ensuring that interventions remain strictly counteractive, the CPT framework seeks to buy users the necessary cognitive resources to pause, reflect, and autonomously form an authentic thought, effectively turning the ethical tension of persuasive design into a mechanism for restoring digital agency.

## 6 Limitations

While this hypothesis and theory article provides a foundational exploration of SDE and CPT, we recognize several theoretical and methodological boundaries that outline necessary future work:

First, our synthesis is derived from an annotated portfolio of three mitigation tools originating from a shared research stream (Kronhardt et al., 2024b,a, 2025b). While this RtD approach is precisely intended to generate intermediate-level knowledge (Bowers, 2012), the resulting concepts of SDE and CPT represent an initial formalization. Because these mitigation tools were developed within a shared research environment, they may naturally exhibit structural overlaps. The structural similarities we identify between the mitigation tools may therefore reflect our own design habits and theoretical priors rather than a mind-independent property. The portfolio cannot, as a methodological limitation, distinguish between these two possibilities. To mature this intermediate-level knowledge into a generalized analytical framework, future empirical work must apply the CPT criteria to a wider corpus of independently developed artifacts.

A further boundary concerns cultural and regulatory context. Our portfolio was developed entirely within a German and European setting, shaped by the GDPR, the Digital Services Act and the Artificial Intelligence Act. This bears directly on our fourth boundary condition, which asks whether an exchange was disclosed and evaluable rather than whether it was favorable. What counts as adequate disclosure, and which commercial persuasion practices are treated as ordinary rather than exploitative, varies across jurisdictions and cultures. We formulate the condition in relative and procedural terms for precisely this reason, but its application will nonetheless require sensitivity to local regulatory baselines and cultural norms, which our portfolio is not positioned to establish.

Further, while our discussion outlines initial boundary conditions for SDE, these require further theoretical stress-testing. This paper focused on Deceptive Patterns, Algorithmic Profiling, and Information Disorder; however, the core mechanism of exploiting psychological vulnerabilities to induce Knowledge-Behavior Gaps applies to adjacent digital harms. Our proposed boundaries therefore need to be applied to these broader contexts to refine the concept's analytical rigor and confirm exactly where SDE begins and ends.

Finally, while CPT theoretically defines success as the facilitation of integrated regulation rather than behavioral compliance with a system-defined goal, effectively measuring this psychological shift remains a methodological challenge. Future empirical work must develop standardized evaluation metrics to operationalize and quantify the restoration of mental self-determination, ensuring that a user's transition away from external regulation can be accurately tracked and assessed in practice.

## 7 Conclusion and future work

As digital environments increasingly shift from neutral toolsets into adversarial ecosystems, traditional paradigms of passive transparency are no longer sufficient to protect users. Rather than treating Deceptive Patterns, Algorithmic Profiling, and Information Disorder as isolated design failures, our retrospective reflection upon an annotated portfolio of mitigation tools proposes viewing them as symptoms of a potentially unified mechanism. We tentatively formalize this mechanism as an intermediate-level conceptual construct: *Systemic Digital Exploitation*. While operating through different modalities, we hypothesize that these harms may share a common architectural root: the targeted exploitation of psychological vulnerabilities to induce a Knowledge-Behavior Gap that actively thwarts user autonomy.

If algorithmic manipulation threatens an individual's fundamental right to mental self-determination—their capacity to freely form independent thoughts (Faraoni, 2023)—then mitigation requires an active, structured defense. To help align the design thinking behind these countermeasures, we propose the *Counteractive Persuasive Technology* framework. Positioned as a bridging concept, CPT connects macro-psychological theory with actionable design practice. By categorizing tools into Capacity-Building (restorative training) and Scaffolding (compensatory real-time support) modes, CPT offers a structured vocabulary for conceptualizing how systems might actively restore user agency. Crucially, this framework explicitly rejects system-defined behavioral goals to avoid the ethical trap of soft paternalism. Instead, it uses persuasion solely to buy users the cognitive resources needed to align actions with their reflective values and foster integrated regulation and holds designers accountable through its two-step design process and required identification of both the SDE they aim to counter and the psychological mechanism through which the exploitation happens.

This mandate also bounds what a CPT asks of the user. Because the framework prescribes no behavioral outcome, there is no regimen to adhere to and no target to meet: the user is asked only for the moment of deliberation the exploitation had removed. Power and simplicity are therefore not in tension here, since a CPT that imposed a substantial ongoing burden would be failing on its own terms, spending the very cognitive resources it exists to restore.

By offering these provisional concepts, our goal is to catalyze community discourse around the boundary conditions of SDE and the structural mechanisms of counter-persuasion. Recognizing that this framework is derived from a specific, curated portfolio of our own design work, future empirical work must validate and refine these concepts by applying the CPT analytical lens to a broader, independently developed corpus of mitigation tools. To outline a roadmap for this future direction, the next step in stress-testing this bridging concept involves a two-pronged approach: analytical and generative validation.

First, analytical validation will be conducted through a systematic literature review of interventions targeting SDE phenomena. By classifying existing tools outside our annotated portfolio, we can map the current landscape of counteractive designs. This step will refine the boundary conditions of SDE and validate the CPT framework's descriptive qualities. To illustrate, we have selected three tools from different research streams as examples:

- The *Iffy or Not* extension (Lim et al., 2026) could be classified as a CPT operating in a *Scaffolding* mode, utilizing Socratic questioning to disrupt the cognitive heuristics associated with Information Disorder.
- Interventions like the *Bad News* game (Roozenbeek and van der Linden, 2019) map onto the *Capacity-Building* mode, employing role-play as an active inoculation strategy to preemptively build resistance against manipulative environments.
- Privacy interventions that intermittently deploy cognitive “boosts” to align browsing behavior with stated privacy goals (Ortloff et al., 2021) directly operationalize CPT's objective of *bridging the Knowledge-Behavior Gap*.

Second, generative evaluation of the framework requires empirical user studies to test its utility as a practical ideation tool. Future research should identify an as-of-yet unaddressed SDE phenomenon and use the CPT framework to develop a novel counteractive intervention. This intervention should then be evaluated against a baseline state to explicitly measure whether the CPT demonstrably increases users' perceived autonomy during decision-making. We further propose measurement of the Knowledge-Behavior Gap itself as a concrete validation criterion for the construct. Since the gap is currently supported by prior literature on each phenomenon rather than by measurement within our own portfolio, future CPT evaluations should capture both terms of the relevant contradiction (for instance, stated privacy concern alongside observed disclosure behavior) before and after exposure to the intervention, and demonstrate that the intervention narrows the distance between them. Establishing whether CPTs measurably close the gap they are theorized to bridge would provide the central empirical test of the mechanism proposed here, and would also supply the operational measure that our fourth limitation identifies as presently lacking.

Ultimately, we offer this intermediate-level knowledge to provide a shared theoretical lens, empowering researchers and practitioners to look beyond isolated silos and collaboratively design practical, ethically sound interventions that restore mental self-determination and user autonomy in hostile digital environments.

## Data Availability

The original contributions presented in the study are included in the article/supplementary material, further inquiries can be directed to the corresponding author.

## Footnotes

1 A well-documented instance is a firmware update distributed by HP Inc. in September 2016, which caused OfficeJet printers already in users' possession to reject third-party and refilled cartridges they had previously accepted. Following public criticism, the manufacturer issued an optional firmware release restoring the earlier behavior. See Meyer, D. (2016), “HP Inc Backtracks On Its Controversial Printer Lockdown”, *Fortune*, 29 September 2016, https://fortune.com/2016/09/29/hp-printer-ink/ (last accessed 12.09.2026).
